# LncRNA SNHG20 promoted proliferation, invasion and inhibited cell apoptosis of lung adenocarcinoma via sponging miR‐342 and upregulating DDX49

**DOI:** 10.1111/1759-7714.13693

**Published:** 2020-10-22

**Authors:** Xiuli Wang, Guomin Gu, Hongge Zhu, Suqiong Lu, Kahaerjiang Abuduwaili, Chunling Liu

**Affiliations:** ^1^ Pulmonary Medicine Department The Affiliated Tumor Hospital of Xinjiang Medical University Urumqi, Xinjiang China

**Keywords:** DDX49, lung adenocarcinoma, miR‐342, proliferation, SNHG20

## Abstract

**Background:**

There is increasing evidence that long non‐coding RNA (lncRNA) small nucleolar RNA host gene 20 (SNHG20) plays an important role in cancer. However, the function of SNHG20 in lung adenocarcinoma is unclear. The aim of our study was to investigate the roles of SNHG20 in lung adenocarcinoma.

**Methods:**

Real‐time quantitative polymerasechain reaction (RT‐qPCR) was used to calculate the expression of SNHG20, miR‐342 and DEAD‐box helicase 49 (DDX49). Dual luciferase reporter gene assay was applied to verify whether miR‐342 binding to SNHG20 and DDX49. The expression correlation between miR‐342 and SNHG20 or DDX49 was assessed using Pearson's correlation analysis.

**Results:**

SNHG20 and DDX49 were overexpressed, while miR‐342 was lowly expressed in lung adenocarcinoma tissues and cell lines. Knockdown of SNHG20 suppressed cell proliferation, invasion and enhanced cell apoptosis. SNHG20 was found to directly bind to miR‐342 and regulate the expression of miR‐342. MiR‐342 directly targeted DDX49 and the expression of miR‐342 had negative connection with DDX49 in lung adenocarcinoma tissues. Knockdown of DDX49 inhibited the progression of lung adenocarcinoma. DDX49 partially restored the functions of SNHG20 in A549 cells.

**Conclusions:**

SNHG20 regulated lung adenocarcinoma cell proliferation, invasion and promoted cell apoptosis via miR‐342/DDX49 axis. Our findings demonstrate that SNHG20/miR‐342/DDX49 axis plays an important role in lung adenocarcinoma, providing a novel insight into the treatment of lung adenocarcinoma.

## Graphical abstract

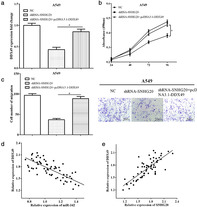

SNHG20 regulated lung adenocarcinoma cell proliferation, invasion and promoted cell apoptosis via miR‐342/DDX49 axis. SNHG20 regulates DDX49 expression via sponging miR‐342 to regulate cell proliferation, migration and apoptosis in lung adenocarcinoma.

## Introduction

Lung cancer (LC) is a common cancer type, and the leading cause of cancer‐related deaths worldwide.^1^ Non‐small cell lung cancer (NSCLC) accounts for 85% of all cases of lung cancer.^2^ Lung adenocarcinoma (LUAD) is a frequent histological subtype of NSCLC that accounts for 40% of all lung cancers.^3^ Although clinical treatment and molecular therapy has been significantly improved, the five‐year overall survival of lung cancer is less than 15%.^4^, ^5^ Thus, elucidating the molecular mechanism of LUAD progression is important to facilitate the development of appropriate methods of diagnosis and treatment.

Long non‐coding RNAs (lncRNAs) are a type of RNA that are longer than 200 nucleotides.^6^ LncRNAs are recognized to play a central regulatory role in biological processes.^7^ More importantly, emerging evidence has revealed that lncRNA exerts its function in various cancers by promoting tumor growth and metastasis.^8^ For example, lncRNA SNHG20 localized at 17q25.2, promoted cell proliferation of laryngeal squamous cell carcinoma via targeting miR‐140.^9^ In addition, SNHG20 enhanced prostate cancer cell proliferation and invasion but decreased cell apoptosis via binding to miR‐6516‐5p/SCGB2A1 axis.^10^ Similarly, SNHG20 promoted cell proliferation and migration, and inhibited cell apoptosis in oral squamous cell carcinoma.^11^ Even in NSCLC, knockdown of SNHG20 suppressed proliferation, motility, and improved apoptosis.^12^ However, the role of SNHG20 in lung adenocarcinoma is still unclear.

MicroRNA (miRNA) is a novel type short non‐coding RNA which is 19–24 nucleotides in length.^13^ MiRNAs have been shown to act as negative regulators regulating gene expression via directly binding to the 3′‐untranslated region (3′‐UTR) at post‐transcriptional level.^14^ Studies have demonstrated that miRNAs usually play carcinogenic or tumor suppressive roles in multiple cancers.^15^ For example, miR‐342 has been reported to significantly decrease cell proliferation, migration and colony formation in hepatocellular carcinoma.^16^ MiR‐342 also caused significant inhibition of the proliferation, colony formation, invasion and migration of breast cancer.^17^

The DExD RNA helicases contains a conserved core consisting of nine conserved motifs that include the characteristic D‐E‐x‐D motif, which confer ATP hydrolysis and RNA unwinding activities, establishing it as RNA helicase.^18^ The DExD RNA helicases, belong to the superfamily 2 of helicases, which participate in almost all aspects of RNA metabolism.^19^ For example, DHX9 has been reported to suppress the anti‐HBV effect of A3B via interacting with APOBEC3B in hepatoma carcinoma cell.^20^ However, the roles of DEAD‐box helicase 49 (DDX49) are still unknown.

## Methods

### Patient specimens

During January 2015 and Department 2018, 63 pairs of lung adenocarcinoma tissues and adjacent nontumor tissue samples were obtained from 63 patients treated by The Affiliated Tumor Hospital of Xinjiang Medical University. Patients who had received anticancer treatment were excluded from this study. The tissues were snap‐frozen in liquid nitrogen and stored at −80°C for future use. The research was approved by the Ethics Committee of The Affiliated Tumor Hospital of Xinjiang Medical University, and the subjects provided written informed consent for their participation in the study.

### Cell culture

Lung adenocarcinoma cell lines A549 and H1299 and a normal epithelial cell line 16HEB were purchased from the American Type Culture Collection (ATCC, Manassas, VA, USA). All cells were cultured in RPMI‐1640 supplemented with 10% fetal bovine serum (FBS, InvivoGen, San Diego, CA), 100 U/mL penicillin and 100 mg/mL streptomycin at 37°C in an atmosphere of 5% CO_2_.

### Cell transfection

The siRNA specifically targeting SNHG20 (siRNA‐SNHG20, 5′‐GCCACUCACAAGAGUGUAUTT‐3′) and the negative control (si‐NC, 5′‐GGATACGGAGTACTATAGC‐3′), siRNA‐DDX49 (5′‐UGGGCCAUCCACCUCCAAUG‐3′) and their negative controls (5′‐TTCTCCGAACGTGTCACGT‐3′) were designed and synthesized by RiboBio (Guangzhou, China). Meanwhile, the miR‐342 mimic (5′‐AGCAGAGGCAGAGAGGCUCAGG‐3′) or the control (5′‐AGGAUGUAUUACCAGUGAUCGG‐3′) were synthesized from GenePharma (Shanghai, China). The oligonucleotides were transfected into A549 cells using Lipofectamine 3000 (Invitrogen, Carlsbad, USA).

### RNA extraction and quantitative real‐time PCR (qRT‐PCR) analysis

The TRIzol Reagent (Invitrogen, Carlsbad, USA) was used for extracting total RNA according to the manufacturer's protocol. The RNA sample was quantified by NanoDrop 2000, and reverse transcribed into complementary DNA (cDNA) using M‐MLV Reverse Transcriptase (Promega, Madison, WI, USA). Followed, the SYBR‐Green Reagent kit (Qiagen, Hilden, Germany) and the SYBR PrimeScript miRNA RT PCR kit (Takara, Dalian, China) was used to detect the mRNA level of SNHG20, DDX49 and miR‐342 on ABI 7500 system (Applied Biosystems, Foster City, CA, USA). The glyceraldehyde‐3‐phosphate dehydrogenase (GAPDH) or small nuclear RNA U6 (U6) were used as endogenous control. The primers were listed as follows: SNHG20 F‐5′‐ATGGCTATAAATAGATACACG‐3′, R‐5′‐GGTACAAACAGGGAGGGA‐3′; DDX49 F‐5′‐CCTACCAGATCGCAGAGCAG‐3′, R‐5′‐ CACGTGTGGTTTCCGAGAGA‐3′; GAPDH F‐5′‐TCCCATCACCATCTTCCAGG‐3′, R‐5′‐GATGACCCTTTTGGCTCCC‐3′; miR‐342 F‐5′‐TCCTCGCTCTCACACAGAAATC‐3′, R‐5′‐TATGGTTGTTCACGACTCCTTCAC‐3′; U6 F‐5′‐AACGAGACGACGACAGAC‐3′, R‐5′‐GCAAATTCGTGAAGCGTTCCATA‐3′.

### Cell proliferation assay

Cell viability was evaluated using 3‐(4,5‐Dimethylthiazol‐2‐yl)‐2,5‐diphenyl‐2Htetrazol‐3‐ium bromide (MTT, Abcam, Shanghai, China) assay. Briefly, A549 cells were placed on a culture plate at a density of 4 × 103 cells per well. After being cultured for 24 hours, 48 hours, 72 hours and 96 hours, the cells were stained with 100 μL sterile MTT at 37°C for a further 4 hours. After the supernatant was discarded and replaced, dimethyl sulfoxide (DMSO, Sigma‐Aldrich, China) was employed to dissolve the formazan crystals for 10 minutes. The absorbance at 490 nm was subsequently evaluated on a microplate reader (Applied Biosystems, Foster City, CA, China). All experiments were repeated three times.

### Transwell assay

Transwell inserts of 8 μm pore size (Merck Millipore, Eschborn, Germany) covered with Matrigel matrix (BD Science, Sparks, MD, USA) were utilized to measure cell invasion. A549 cells were added to the upper chamber without FBS, while the lower chamber was filled with 600 μL of complete medium that contained 20% FBS. After incubation, 20% methanol and 0.1% crystal violet was applied to fix and stain the invaded cells. Meanwhile, the noninvaded cells were removed using a cotton swab. Finally, invasion ability was calculated by counting the number of invasive cells in random three fields on a microscopy (Applied Biosystems, Foster City, CA, USA).

### Cell apoptotic assay

Cell apoptosis was detected using the fluorescein isothiocyanate (FITC) Annexin V‐propidium iodide (PI) Kit (Beyotime, Jiangsu, China). We collected the A549 cells and these were fixed using 75% ethanol. Away from light, the cells were stained with 25 μg/mL Annexin V‐FITC and 40 μg/mL propidium iodide (Invitrogen, Carlsbad, CA, USA). Cell apoptosis rate was analyzed on a FACSorter flow cytometer (Applied Biosystems, Foster City, CA, USA) according to the manufacturer's protocol.

### Dual‐luciferase reporter assay

StarBase prediction software and TargetScan were employed to verify the predictive binding site between miR‐342 and SNHG20 or DDX49. The SNHG20 or DDX49 3′‐UTR fragments containing miR‐342 binding sites and their mutants were cloned into pGL3‐ vectors (Realgene, Nanjing, China), referred as SNHG20‐WT, SNHG20‐MUT, DDX49‐WT, and DDX49‐MUT. The wild‐ or mutant type and miR‐342 mimic or miR‐NC were cotransfected into A549 cells with Lipofectamine 3000 (Invitrogen, Carlsbad, USA). Then, 48 hours after transfection, the cells were harvested and used for the luciferase activity assay.

### Statistical analysis

The data in this study are shown as mean ± standard deviation (SD). GraphPad Prism 5 V5.01 software (La Jolla, CA, USA) and SPSS 21.0 software (IBM, Somers, NY, USA) were conducted to carry out the statistical analysis. Student's *t*‐test or one‐way analysis of variance were used to compare the difference between two or more groups. Pearson's correlation analysis was applied to detect the correlations between miR‐342 and SNHG20 or DDX49. A *P*‐value <0.05 was considered statistically significant.

## Results

### Knockdown of SNHG20 inhibited cell proliferation, invasion, and promoted cell apoptosis of A549 cells

**Figure 1:**
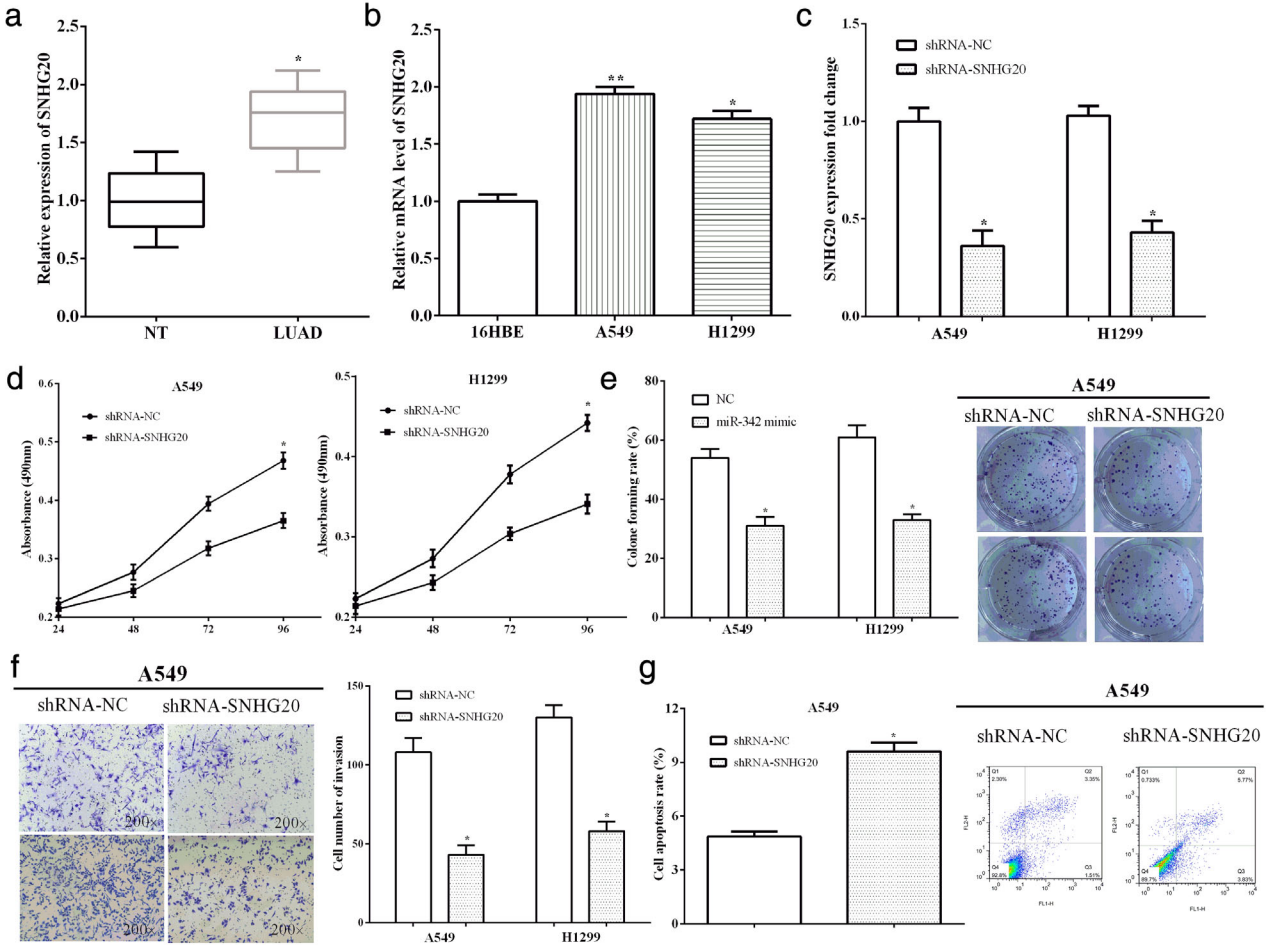
Knockdown of SNHG20 inhibited cell proliferation, invasion, and promoted cell apoptosis of A549 cells. (**a**) SNHG20 was overexpressed in lung adenocarcinoma tissues compared with the paracancerous tissues. (**b**) SNHG20 expression was also higher in lung adenocarcinoma cell lines A549 and H1299 than normal epithelial cell line 16HBE. (**c**) shRNA‐SNHG20 downregulate the expression of SNHG20 in A549 and H1299 cells. (**d**) Cell invasion was reduced after silencing the SNHG20 in A549 and H1299 cells. (**e**) Cell proliferation was also decreased by transfecting of shRNA‐SNHG20 compared to control in A549 and H1299 cells. (**f**) Cell apoptosis was enhanced by shRNA‐SNHG20 versus negative control in A549 cells.

The mRNA level of SNHG20 was calculated in lung adenocarcinoma tissue samples and paracancerous tissue samples. As expected, the expression of SNHG20 was overexpressed in lung adenocarcinoma tissues compared with the paracancerous tissues (*P* < 0.05) (Fig 1a). The clinicopathological characteristics of the patients are shown in Table 1. The expression of SNHG20 is related to TNM stage, lymph‐node metastasis and Ki67, while it has no relationship with gender and age. In addition, SNHG20 has a tendency to correlate with tumor size.

**Table 1:** The expression of SNHG20 and clinicopathological features in 63 lung adenocarcinoma

Clinicopathological features	Cases (*n* = 63)	SNHG20 expression		*P*‐value*
		35 High (%)	28 Low (%)	
Gender				0.910
Male	31	17 (54.8)	14 (45.2)	
Female	32	18 (56.3)	14 (43.7)	
Age (years)				0.397
≤60	30	15 (50.0)	15 (50.0)	
>60	33	20 (60.6)	13 (39.4)	
Tumor size (mm)				0.070
≤5.0	35	23 (65.7)	12 (34.3)	
>5.0	28	12 (42.9)	16 (57.1)	
TNM stage				0.022*
I–II	37	25 (67.6)	12 (32.4)	
III–IV	26	10 (38.5)	16 (61.5)	
Lymph node metastasis				0.015*
0–2	39	26 (66.7)	23 (33.3)	
>2	24	9 (37.5)	15 (62.5)	
Ki67				0.040*
Yes	36	24 (66.7)	12 (33.3)	
No	27	11 (40.7)	16 (59.3)	

* *P*‐values are calculated with Chi‐square test.

Moreover, SNHG20 expression was also higher in lung adenocarcinoma cell lines A549 (*P* < 0.05) and H1299 (*P* < 0.05) than the normal epithelial cell line 16HBE (Fig 1b). To investigate the important roles of SNHG20 in lung adenocarcinoma, shRNA‐SNHG20 was used to downregulate the SNHG20 in A549 and H1299 cells, and the knockdown efficiency was measured using RT‐qPCR, as shown in Fig 1c.

MTT, transwell and flow cytometry assays were employed to detect cell proliferation, invasion and apoptosis. We found that cell proliferation was reduced after silencing the SNHG20 in A549 (*P* < 0.05) and H1299 (*P* < 0.05) cells (Fig 1d). Moreover, silencing of SNHG20 inhibited the clone formation ability of A549 (*P* < 0.05) cells and H1299 (*P* < 0.05) cells (Fig 1e). Cell invasion was also decreased by transfection of shRNA‐SNHG20 compared to control in A549 (*P* < 0.05) and H1299 (*P* < 0.05) cells (Fig 1f). In contrast, cell apoptosis was enhanced by shRNA‐SNHG20 versus negative control (*P* < 0.05) (Fig 1g). All the findings showed that knockdown of SNHG20 inhibited the progression of lung adenocarcinoma.

### SNHG20 could directly bind to miR‐342 and regulate miR‐342 expression

**Figure 2:**
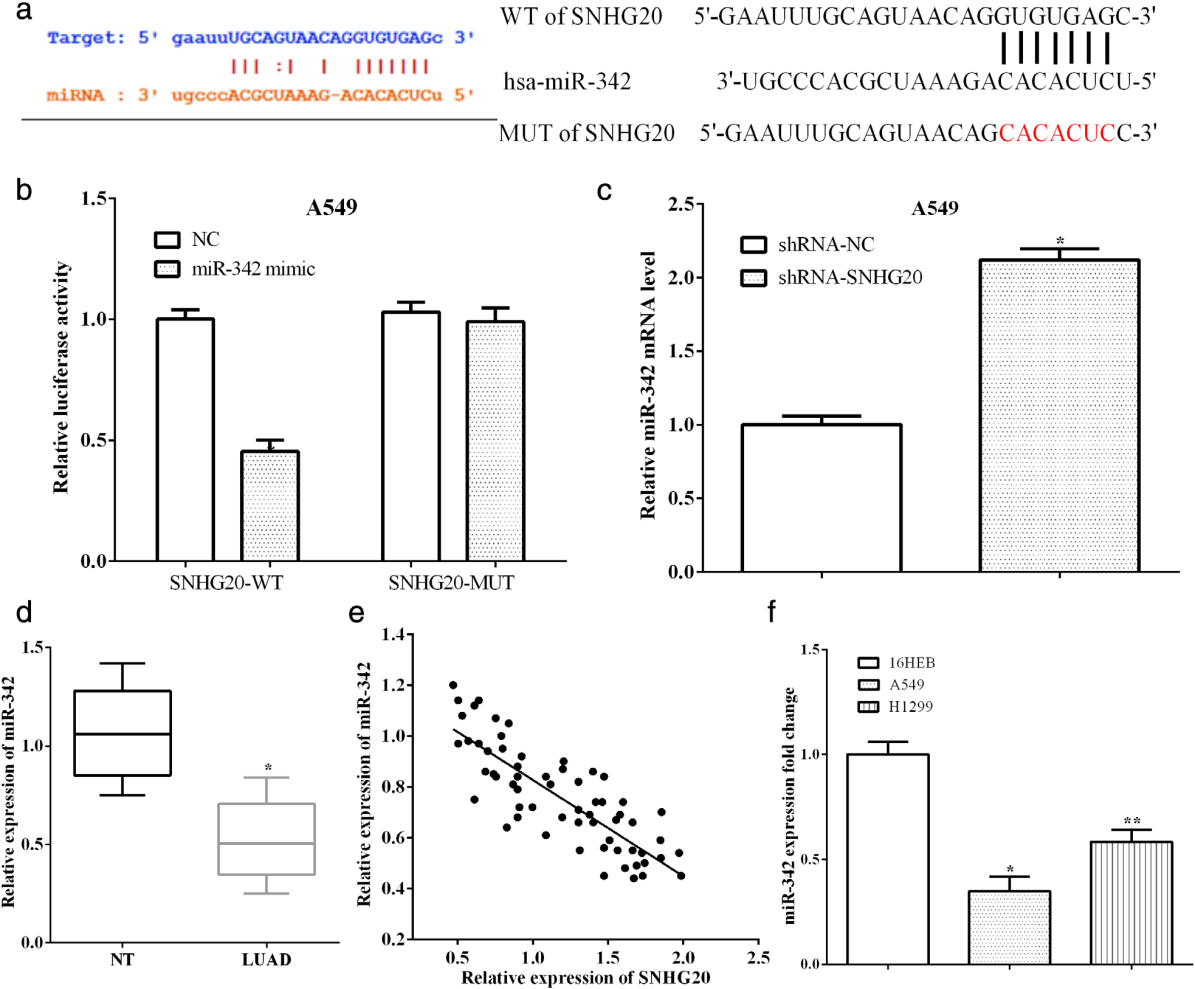
SNHG20 directly bind to miR‐342 and regulate the expression of miR‐342. (**a**) StarBase prediction software predicted the SNHG20 containing a possible binding site of miR‐342. (**b**) MiR‐342 mimic reduced the luciferase activity of wild‐type SNHG20, while it had no effect on mutant SNHG20. (**c**) The expression of miR‐342 was enhanced by knocking down SNHG20. (**d**) MiR‐342 was lowly expressed in lung adenocarcinoma tissues compared with paracancerous tissues. (**e**) The expression of SNHG20 has a negative connection with miR‐342 expression in lung adenocarcinoma tissues. (**f**) In addition, miR‐342 expression was also lower in lung adenocarcinoma cell lines A549 and H1299 than that of 16HEB cells.

StarBase prediction software predicted the SNHG20 containing a possible binding site of miR‐342 (Fig 2a). To verify whether SNHG20 could directly bind to miR‐342, the binding sequences on SNHG20 were mutated and a luciferase reporter assay performed. The wild‐type or mutant SNHG20 and miR‐342 mimic were cotransfected into A549 cells. As expected, miR‐342 mimic reduced the luciferase activity of wild‐type SNHG20 (*P* < 0.05), while it had no effect on mutant SNHG20 (*P* > 0.05) (Fig 2b). In addition, when SNHG20 was knocked down in A549 cells, the expression of miR‐342 was enhanced, as shown in Figure 2c (*P* < 0.05).

The expression of miR‐342 in tissues and cell lines were assessed by RT‐qPCR. MiR‐342 was lowly expressed in lung adenocarcinoma tissues compared with paracancerous tissues (*P* < 0.05) (Fig 2d). The expression of SNHG20 had a negative connection with miR‐342 expression in lung adenocarcinoma tissues (*P* < 0.05) (Fig 2e). In addition, miR‐342 expression was also lower in lung adenocarcinoma cell lines A549 (*P* < 0.05) and H1299 (*P* < 0.05) than that of 16HEB cells (Fig 2f). These results indicated that SNHG20 directly binds to miR‐342 in A549 cells.

### MiR‐342 regulated the expression of DDX49 through directly targeting its 3′‐UTR

**Figure 3:**
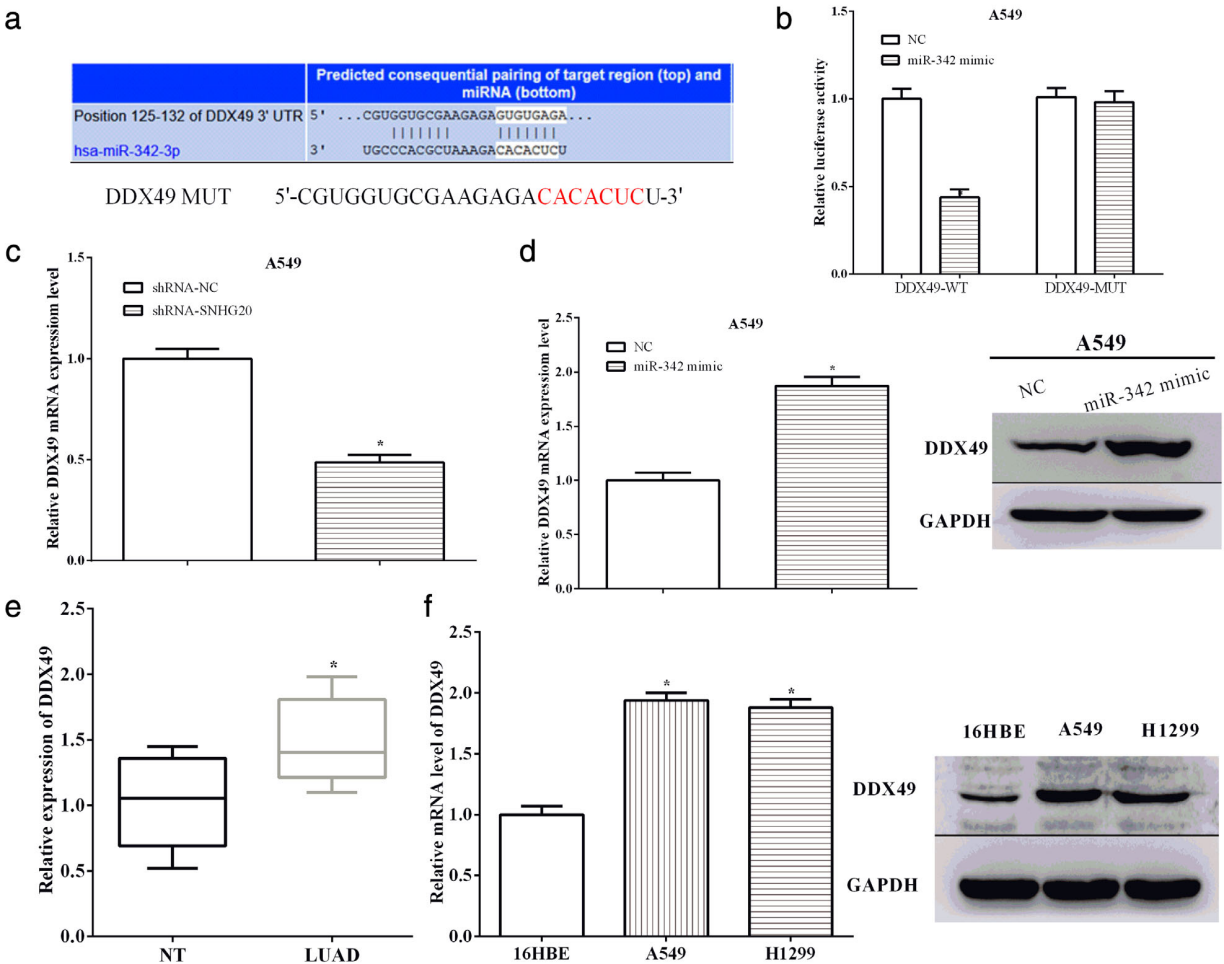
MiR‐342 regulated the expression of DDX49 through directly targeting its 3′‐UTR. (**a**) TargetScan predicted that DDX49 has a direct binding sequences of miR‐342. (**b**) The luciferase reporter gene assay was performed. (**c**) Knockdown of SNHG20 decreased the expression of DDX49 in A549 cells. (**d**) Overexpression of miR‐342 increased the mRNA and protein of DDX49 in A549 cells. (**e**) DDX49 was upregulated in lung adenocarcinoma tissues compared with paracancerous tissues. (**f**) The mRNA and protein levels of DDX49 were also higher in A549 and H1299 cells compared to 16HBE cells.

TargetScan predicted that DDX49 has a direct binding sequences of miR‐342. To verify that miR‐342 directly binds to DDX49, the predicted binding sequences were mutated from GUGUGAGA to CACACUC, and we then performed a luciferase reporter gene assay (Fig 3a). Not surprisingly, miR‐342 mimic reduced the luciferase activity of wild‐type DDX49 (*P* < 0.05), while miR‐342 mimic had no effect on mutant DDX49 (*P* > 0.05) (Fig 3b). The expression of DDX49 was calculated after altering SNHG20 or miR‐342. As expected, knockdown of SNHG20 decreased the expression of DDX49 in A549 cells (*P* < 0.05) (Fig 3c). On the contrary, overexpression of miR‐342 increased the mRNA and protein levels of DDX49 in A549 cells (*P* < 0.05) (Fig 3d).

To explore the roles of DDX49, the expression of DDX49 was evaluated in tissues and cell lines. We found that DDX49 was upregulated in lung adenocarcinoma tissues compared with paracancerous tissues (*P* < 0.05) (Fig 3e). In addition, the mRNA and protein levels of DDX49 were also higher in A549 (*P* < 0.05) and H1299 (*P* < 0.05) cells compared to 16HBE cells (Fig 3f).

### Knockdown of DDX49 inhibited cell proliferation, invasion and promoted cell apoptosis in A549 cells

**Figure 4:**
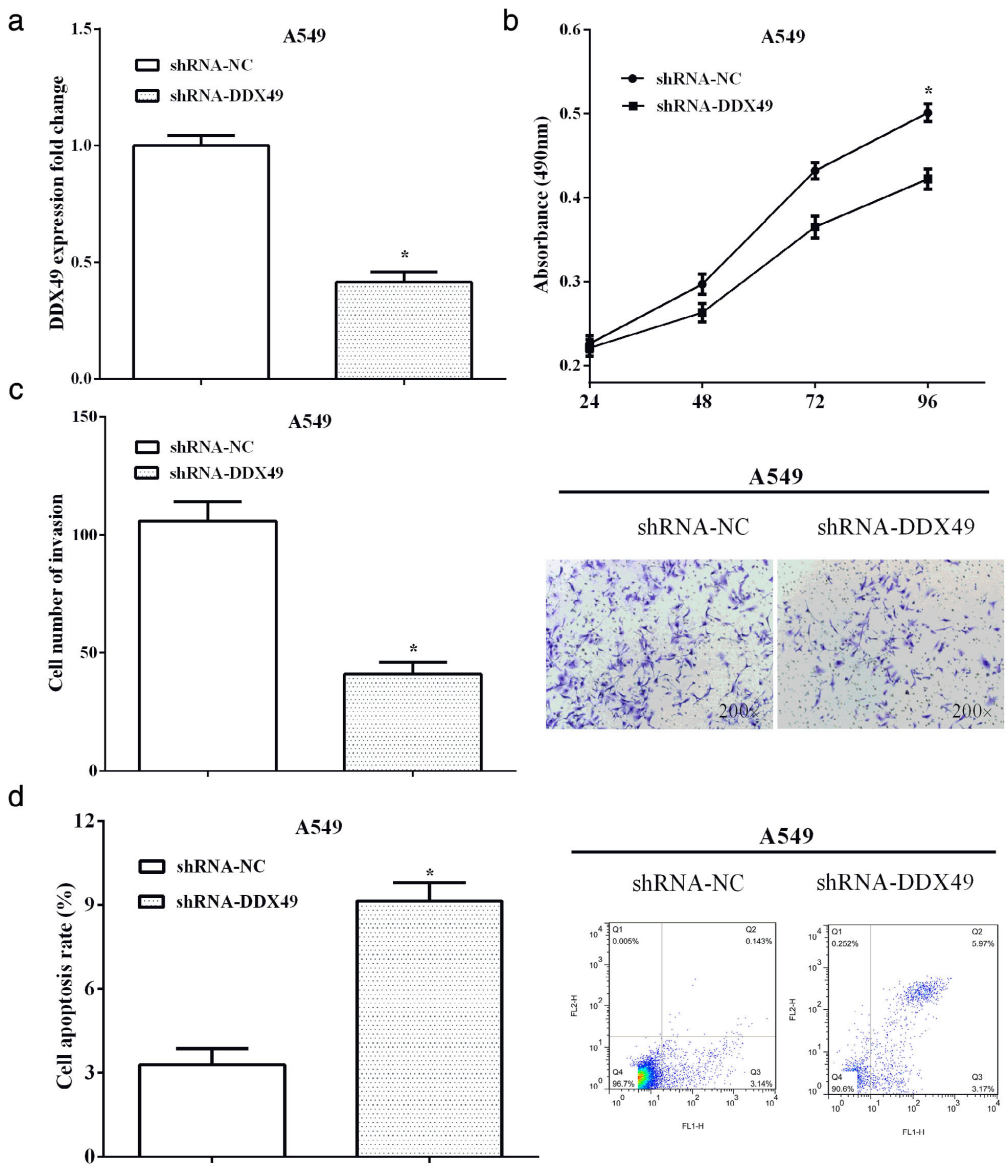
Knockdown of DDX49 inhibited cell proliferation, invasion and promoted cell apoptosis in A549 cells. (**a**) shRNA‐DDX49 was used to knockdown DDX49 in A549 cells. (**b**) Cell viability of the DDX49 silencing group was significantly increased compared with the control group. (**c**) Cell invasion was reduced by silencing DDX49 in comparison with negative control. (**d**) Silencing of DDX49 enhanced cell apoptosis compared with negative control.

To investigate the functions of DDX49, shRNA‐DDX49 was used to knockdown DDX49 in A549 cells, as shown in Figure 4a (*P* < 0.05). Cell viability of the DDX49 silencing group was significantly increased compared with the control group (*P* < 0.05) (Fig 4b). Moreover, cell invasion was also reduced by silencing DDX49 in comparison with the negative control (*P* < 0.05) (Fig 4c). In contrast, silencing of DDX49 enhanced cell apoptosis compared with the negative control (*P* < 0.05) (Fig 4d).

### SNHG20 regulated cells growth, mobility, and induced apoptosis via regulating miR‐342/ DDX49

**Figure 5:**
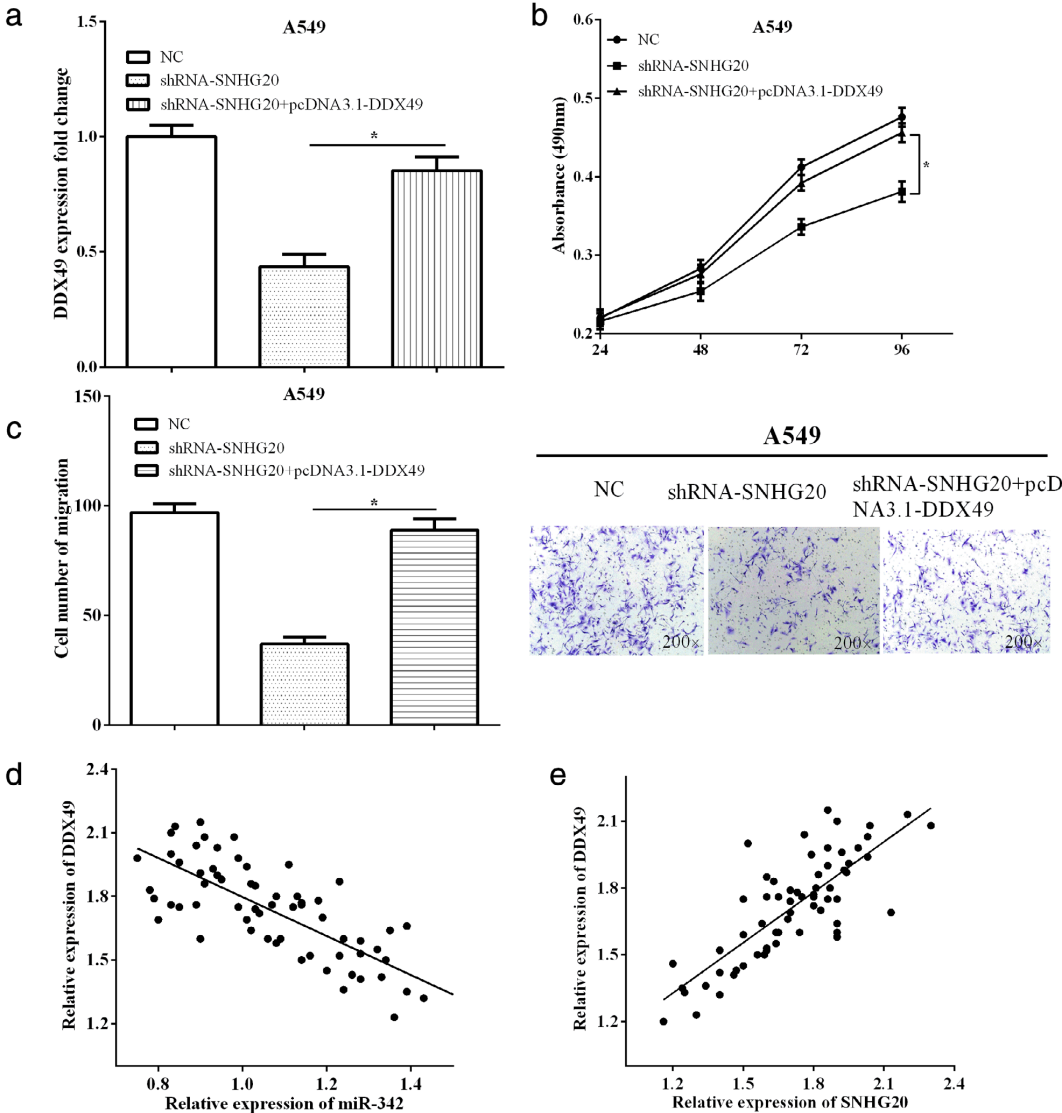
SNHG20 regulated cells growth, mobility, and induced apoptosis via regulating miR‐342/ DDX49. (**a**) The decrease of DDX49 by shRNA‐SNHG20 was abolished by overexpression of DDX49. (**b**) pcDNA3.1‐DDX49 recovered partially effect of silencing SNHG20 on cell proliferation. (**c**) pcDNA3.1‐DDX49 partially reversed the functions of SNHG20 on cell invasion. (**d**) DDX49 was found to have negative connection with miR‐342 expression. (**e**) DDX49 was positively correlated to SNHG20 expression in lung adenocarcinoma.

In order to explore the interaction between SNHG20, miR‐342 and DDX49 in lung adenocarcinoma, the expression of DDX49 was calculated when cotransfected with shRNA‐SNHG20 and pcDNA3.1‐DDX49 in A549 cells. The results demonstrated that the decrease of DDX49 by shRNA‐SNHG20 was abolished by pcDNA3.1‐DDX49 (*P* < 0.05) (Fig 5a). Functionally, pcDNA3.1‐DDX49 recovered partially the effect of silencing SNHG20 on cell proliferation (*P* < 0.05) (Fig 5b). Similarly, overexpression of partially reversed the functions of SNHG20 on cell invasion (*P* < 0.05) (Fig 5c). DDX49 was found to have negative connection with miR‐342 expression (*P* < 0.05) (Fig 5d), and was positively related to SNHG20 expression in lung adenocarcinoma (*P* < 0.05) (Fig 5e). All the findings indicated that SNHG20 regulated cells growth, mobility, and induced apoptosis via regulating miR‐342/DDX49.

## Discussion

In recent years, there has been increasing evidence that lncRNA acts as competitive endogenous RNA of miRNA to regulate cancer progression.^21^, ^22^, ^23^ Long non‐coding RNA SNHG20, a member of lncRNAs, has been reported to be frequently abnormally expressed in multiple cancers, including cervical cancer, esophageal squamous cell carcinoma, gastric cancer and oral squamous cell carcinoma.^24^, ^25^, ^26^, ^27^ SNHG20 has been shown to be significantly upregulated in breast cancer, and participated in cell proliferation, invasion and migration through miR‐495.^28^ Similarly, SNHG20 enhanced cell proliferation and invasion via the miR‐140/ADAM10 axis in cervical cancer.^29^ Consistent with these findings, we found that SNHG20 was remarkably overexpressed in lung adenocarcinoma tissues and cell lines compared with paracancerous tissues and normal cells. Knockdown of SNHG20 inhibited cell viability and invasion in A549 cells. In addition, SNHG20 promoted bladder cancer cell proliferation, colony formation, migration and invasion, and induced cell apoptosis, and SNHG20 was associated with advanced clinical stage, lymph node metastasis, and reduced patient survival rate of bladder cancer patients.^30^ SNHG20 has been reported to cause a significant reduction in cancer cell apoptosis in A549 cells, which was consistent with the findings in non‐small cell lung cancer.^31^ Through tissue verification, the expression of SNHG20 in lung adenocarcinoma has been shown to be related to TNM stage, lymph‐node metastasis and the expression of Ki67. The expression of SNHG20 has a trend of relationship with tumor size, which we speculate is due to the small sample size.

It has been demonstrated that miRNAs act as oncogenes or tumor suppressors in cancers.^32^ MiR‐342 has been reported to target CBX2 and inhibit cell proliferation, metastasis and invasion of ovarian cancer cells.^33^ Similarly, miR‐342 has been shown to suppress breast cancer cell growth, migration and invasion.^17^ MiR‐342 has been demonstrated to act as ceRNA of lncRNAs, including LINC00634, RNA LINC00460 and lncRNA ZEB1‐AS1.^34^, ^35^, ^36^ We discovered that SNHG20 directly binds to miR‐342 after performing a luciferase reporter gene assay. Moreover, knockdown of SNHG20 enhanced the expression of miR‐342 in A549 cells. The expression of SNHG20 has a negative connection with miR‐342 expression in lung adenocarcinoma tissues.

DDX49 was found to be a target gene of miR‐342, verified by luciferase activity reporter gene assay. DExD RNA helicases play great roles in cancers. Loss of DDX39B has been reported to promote resistance to alkylating chemotherapy in glioblastoma cells.^37^ Similarly, DDX19 impaired type I interferon production, resulting in suppression of encephalomyocarditis virus replication.^38^ In this study, we discovered that DDX49 was overexpressed in lung adenocarcinoma tissues and cell lines in comparison with paracancerous tissues and normal cells. Knockdown of DDX49 suppressed A549 cell viability, invasion and promoted cell apoptosis. Furthermore, silencing of SNHG20 or overexpression of miR‐342 inhibited the expression of DDX49 in A549 cells. However, due to lack of tissues, immunohistochemistry was not performed, and this has been included in the deficiency.

In conclusion, SNHG20 was found to play an oncogenic role in the regulation of lung adenocarcinoma cell proliferation, invasion and apoptosis through targeting miR‐342/DDX49 axis. SNHG20 regulated lung adenocarcinoma progression, indicating that SNHG20 may be a biomarker of lung adenocarcinoma.

## Disclosure

The authors declare that there are no conflicts of interest.
